# Pandemic Influenza Detection by Electrically Active Magnetic Nanoparticles and Surface Plasmon Resonance

**DOI:** 10.1109/TNANO.2011.2157936

**Published:** 2011-05-27

**Authors:** Tracy L. Kamikawa, Malgorzata G. Mikolajczyk, Michael Kennedy, Lilin Zhong, Pei Zhang, Emma B. Setterington, Dorothy E. Scott, Evangelyn C. Alocilja

**Affiliations:** 1 U.S. Food and Drug AdministrationCenter for Biologics Evaluation and Research Bethesda MD 20892 USA; 2 Department of Biosystems and Agricultural EngineeringMichigan State University East Lansing MI 48824 USA

**Keywords:** Biosensor, direct-charge transfer, electrically active, immunomagnetic, influenza

## Abstract

Influenza A virus (FLUAV), the causative agent of influenza infection, has received extensive attention due to the recent swine-origin H1N1 pandemic. FLUAV has long been the cause of annual epidemics as well as less frequent but more severe global pandemics. Here, we describe a biosensor utilizing electrically active magnetic (EAM) polyaniline-coated nanoparticles as the transducer in an electrochemical biosensor for rapidly identifying FLUAV strains based on receptor specificity, which will be useful to monitor animal influenza infections and to characterize pandemic potential of strains that have transmitted from animals to humans. Pandemic potential requires human-to-human transmissibility, which is dependent upon FLUAV hemagglutinin (HA) specificity for host glycan receptors. Avian FLUAV preferentially bind to α2,3-linked receptors, while human FLUAV bind to α2,6-linked receptors. EAM nanoparticles were prepared by synthesizing aniline monomer around gamma iron (III) oxide (γ-Fe_2_O_3_) cores, yielding 25–100-nm diameter nanoparticles that were structurally characterized by transmission electron microscopy and electron diffraction. The EAM nanoparticles were coated with monoclonal antibodies specific to H5N1 (A/Vietnam/1203/04). Specificity of binding between glycans and H5 was demonstrated. The biosensor results were correlative to supporting data from a surface plasmon resonance assay that characterized HA/glycan binding and α-H5 antibody activity. This novel study applies EAM nanoparticles as the transducer in a specific, portable, easy-to-use biosensor with great potential for disease monitoring and biosecurity applications.

## I. Introduction

Influenza A virus (FLUAV) is an acute viral disease agent of the respiratory tract, which is classified as a genus of the *Orthomyxoviridae* family [1]. Millions of people worldwide are affected annually by FLUAV. The 2009 H1N1 pandemic originated from a swine FLUAV that has been circulating in pig herds for two decades and demonstrates how quickly a FLUAV with human-to-human transmissibility can spread worldwide [2]. Each strain of FLUAV is named and characterized by its hemagglutinin (HA) and neuraminidase (NA) serotype [3]. The extent of infection into the host organism is determined by the viral surface glycoprotein HA, which mediates FLUAV host specificity and host cell entry [3], [4]. Of the sixteen known HA and nine known NA serotypes, only three HA and two NA have adapted sufficiently to become pandemic in humans, resulting in the historical pandemics of H1N1 in 1918 and 2009, H2N2 in 1957, and H3N2 in 1968. All serotypes of FLUAV circulate in the avian population, leading to the common belief that birds act as the main reservoir for FLUAV [3].

Influenza viruses are subject to antigenic drifts associated with seasonal epidemics, which are responsible for 36 000 deaths and 2 00 000 hospitalizations in the U.S. annually, leading to costs for the nation of over $10 billion [5]. In antigenic drift, the HA undergoes relatively minor changes that result from the selection of mutant viruses by antibodies generated against the prominent HA antigenic type currently circulating in the human population [6]. Of even greater concern are FLUAV pandemics, or global disease outbreaks, which are due to antigenic shifts and result in high mortality rates [6], [7]. The most virulent of these was the 1918–1919 outbreak of H1N1, known as the “Spanish flu,” which resulted in 20–40 million deaths worldwide [8]. Pandemics occur when the genomic RNA segment encoding HA is replaced, allowing FLUAV strains to rapidly adapt from one animal species to another. FLUAV lends itself to such reassortment because the segmented nature of its genome allows for the exchange of entire genes between different viral strains.

Human-to-human transmission of FLUAV infections, and thus pandemic potential, is dependent upon FLUAV HA receptor specificity for host cell glycan receptors with terminal sialic acids (SAs). Avian FLUAV preferentially bind to SAs connected to galactose by α2,3 linkages on lower respiratory tract ciliated cells, whereas human FLUAV preferentially bind to α2,6-linked SAs on nonciliated cells found in the nose and throat. Due to these specificities, there is concern that a pandemic avian flu could achieve human infectivity and transmissibility via antigenic shifting, and there is interest in modeling these receptors using glycan microarrays to better understand HA preferences [3], [9].

Conventional virological methods for influenza virus analysis are well established [10]. The “gold standard” for virus detection is viral isolation culture with immunocytological confirmation of viral antigen. Time to report including sample preparation and testing time is approximately 12–14 days. Other common virus-testing procedures include complement fixation, hemagglutinin-inhibition, and polymerase chain reaction, which require several hours to one day for culture and detection. Commercial diagnostic test kits directly detect influenza A or B virus-associated antigens or enzyme in throat swabs, nasal swabs, or nasal washes. The majority of these tests use anti-influenza antibodies to detect viral antigens, with only a fraction differentiating between influenza A and B. Based on limited published data comparing performance of these commercial diagnostic test kits to conventional virological methods for detecting novel H1N1 virus in clinical specimens, sensitivity of commercial kits ranged from 10–70% [11]. Time to report is typically 30 min [10]. ZStatFlu, manufactured by Zyme Tx, (Oklahoma City, OK) is an NA assay that achieves specificity using modified SA. The test has a specificity of 98.7% but a poor sensitivity of 62.2% as reported by the manufacturer [12]. While rapid test kits for FLUAV detection are commercially available, there is a need for a biosensor technology that decreases definitive turnaround time and offers quantitative results as opposed to subjective color change assessments. A biosensor is the integration of a biological component with an electronic, electrochemical, optical, or acoustic transducer, with the intention of quantifying a physiological or biochemical change in terms of an electrical response [13]–[21]. Descriptions of a wide range of biosensor types can be found in the literature. The conclusion to be drawn from currently available rapid assay techniques is a lack of binding partner novelty; most assays depend on detecting influenza viruses by interactions with influenza-specific antibodies. While the SA receptor has been investigated in NA binding, there lacks a biosensor technology that exploits influenza HA specificity for host SA receptors, which is the cornerstone of viral infectivity. The biosensor presented here is novel in its binding between SA receptors and influenza HA, which fills a gap in previous implementations and offers in-field characterization of influenza host range, which depends on SA specificity.

Conductive polymers such as polyaniline, polypyrrole, polyacetylene, and polythiophene are receiving interest in current research applications due to their combined properties as both conductors of electricity and organic compounds. This combination means that conductive polymers can offer the mechanical flexibility and robustness of a plastic while also providing the electrical conductivity of a metal. Polyaniline is of particular interest for its environmental stability and simple and inexpensive synthesis. Undoped polyaniline is insulating, while the addition of a protic solvent such as hydrochloric acid (HCL) or sulfuric acid, which can release protons, yields a conducting form of polyaniline with an up to ten order of magnitude increase in conductivity [22], [23]. Polyaniline is applicable in various platforms, including batteries, fuel cells, electrodes, and biosensors, largely due to its ability for efficient electronic charge transfer [24]–[27]. Magnetic polyaniline nanoclusters have been described in the literature with various combinations of magnetic cores and doping agents. Magnetic core materials include iron (II, III) oxide, hydroxyl iron, and Li Ni Ferrite, with HCL, phosphoric acid, and toluene as doping agents [28]–[33]. These nanostructures are attractive for their light weight, mechanical strength, and other magnetic properties [34].

The Biacore 3000 system utilizes surface plasmon resonance (SPR) to measure changes in mass at a biospecific surface that result from the interactions of interest. Biomolecular recognition elements recognize and capture analyte in a liquid sample producing a local increase in refractive index at a thin metal film surface. Optical means then accurately measure the refractive index increase that is proportional to the concentration of target in the sample [35], [36]. The label-free, real-time system offers high sensitivity [37]. Schofield and Dimmock [38] first reported the use of SPR for influenza virus detection. The sensor chip was coated with anti-influenza monoclonal antibody, and influenza virus was injected into the flow system. Binding affinity with the surface antibody was monitored, and dissociation and association rate constants were comparable to those from an affinity enzyme-linked immunosorbent assay [38]. SPR has also been used to study the interaction between FLUAV HA and its cell surface receptor SA using a sensitive microscale binding assay [39]. Bromelain-cleaved HA rosettes showed tight binding to a fetuin-derivatized sensor surface. We report relevant results from an SPR (Biacore)-based assay, which studied the specificity and avidity of HA/glycan binding as an initial step in the design of a functional polymer-based biosensor.

**Fig. 1.**
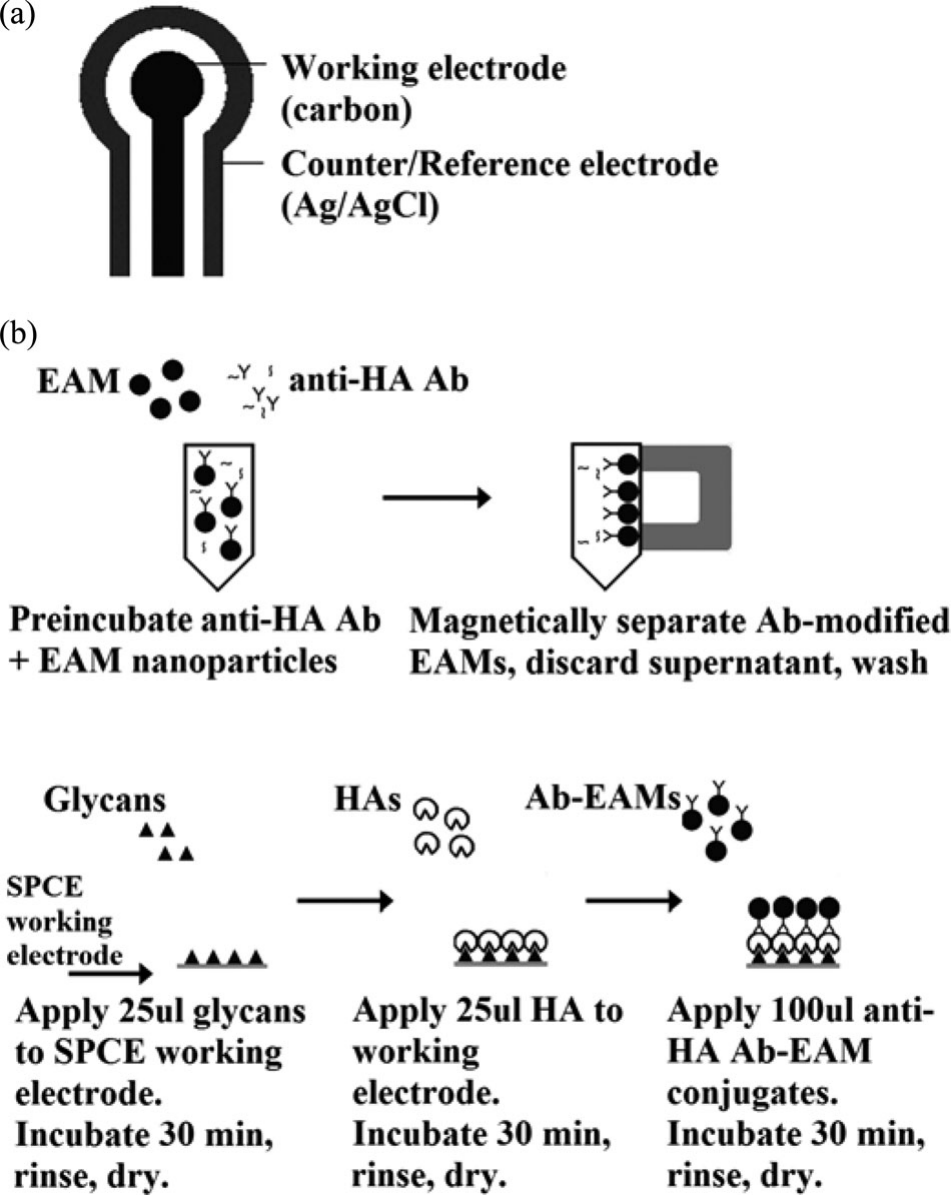
Sensor components and setup. (a) Sensor is composed of two electrodes: the carbon working electrode and the silver/silver chloride counter/reference electrode. (b) Step-wise biosensor setup.

In this experimental study, we investigate the novel application of electrically active magnetic (EAM) polyaniline nanostructures as the transducer in a biosensor for the rapid detection of emerging pandemic FLUAV strains. To our knowledge, such an application has not yet been reported. We propose a biosensor that utilizes EAM nanoparticles on a screen-printed carbon electrode (SPCE) platform. SPCEs are applicable for *in situ* monitoring and are attractive for their high sensitivity, portability, and affordability. Each strip consists of a carbon working electrode and silver reference electrodes printed on low-cost plastic (see Fig. 1). Screen printing offers reproducibility and reliability, and their single-use function offers on-site applicability [40], [41]. This paper presents an efficient and valuable technique using a sequence of magnetic separation, direct electrical detection, and immunochemistry for development of a direct-charge transfer biosensor for rapid, sensitive, and specific detection of FLUAV HA. Here, we investigate binding between purified HA recombinant from baculovirus and purified synthetic glycans with reported HA specificities, as proof-of-concept, on the biosensor platform.

## II. Materials and Methods

### A. Chemicals and Reagents

HBS-P buffer, 10 mM glycine pH 2.5, and 50 mM NaOH were purchased from GE Healthcare, Piscataway, NJ. Avidin/Biotin blocking kit was purchased from Vector Laboratories, Inc., Burlingame, CA. Iron (III) oxide (γ-Fe_2_O_3_) nanopowder, aniline monomer, HCl, ammonium persulfate, methanol, diethyl ether, hydrogen tetrochloroaurate (III) trihydrate, sodium citrate dehydrate, glutaraldehyde, Polysorbate-20 (Tween-20), phosphate buffered saline (PBS), trizma base, casein, sodium phosphate (dibasic and monobasic), and streptavidin were purchased from Sigma-Aldrich, St. Louis, MO. All solutions and buffers used in the biosensor study were prepared in deionized (DI) water (from Millipore Direct-*Q* system) as follows: PBS buffer (10 mM PBS, pH 7.4), wash buffer (10 mM PBS, pH 7.4, with 0.05% Tween-20), phosphate buffer (100 mM phosphate buffer, pH 7.4), casein blocking buffer (100 mM Tris–HCl buffer, pH 7.6, with 0.1% w/v casein), and glycine blocking buffer (67 μM glycine in 10 mM PBS, pH 7.4).

### B. Glycans, HA, and Antibodies

The carbohydrate compounds CT/Sda, 3’SLe^*x*^, and 3’SLN were provided by the Carbohydrate Synthesis/Protein Expression Core of The Consortium for Functional Glycomics funded by the National Institute of General Medical Sciences grant GM62116. The following reagent was obtained through the NIH Biodefense and Emerging Infections Research Resources Repository, NIAID, NIH: H5 Hemagglutinin (HA) Protein from Influenza Virus, A/Vietnam/1203/04 (H5N1), Recombinant from baculovirus, NR-660. The following reagent was obtained through the NIH Biodefense and Emerging Infections Research Resources Repository, NIAID, NIH: Monoclonal Anti-Influenza Virus H5 Hemagglutinin (HA) Protein (VN04–2), A/Vietnam/1203/04 (H5N1), (ascites, Mouse), NR-2728. Anti-H3, A/Shandong/9/93 (H3N2), was purchased from Prospec Protein Specialists (Rehovot, Israel).

### C. SPR (Biacore) Assay Design and Chip Preparation

HA/glycan pairs were chosen based on previous analysis of HA receptor specificities by glycan microarrays [3], [9]. Biotinylated glycans were diluted to 1 μg/ml in Biacore HBS-P buffer and 8 μl were injected over a Biacore Streptavidin chip at 10 μl/min. Glycans were immobilized to saturation at approximately 300 resonance units (RU). H5 HA (Vietnam) at 140 nM was incubated with a serial dilution of α-H5 monoclonal antibody (shown to be neutralizing for H5 HA in standard hemagglutination inhibition assays) or a 1:250 dilution of α-H3 monoclonal antibody for 10 min at 25 °C and injected over the glycan chip surface to investigate the ability of the antibodies to neutralize the H5/glycan binding. Binding was assessed by an increase in RU. After 30-min dissociation time, the glycan surface was regenerated with 30 s of 10 mM glycine pH 2.5 and 1 min of 50 mM NaOH at 100 μl/min. The ability of the α-H5 monoclonal antibody to bind to the H5/glycan complex was also investigated. H5 at 140 nM was injected over the immobilized glycans for 10 min at 5 μl/min. After 1-min dissociation and no regeneration, α-H5 monoclonal antibody was injected over the H5/glycan complex for 5 min at 5 μl/min.

### D. Gold Nanoparticle Synthesis

Gold nanoparticles (AuNPs) were necessary to amplify the response current, improve the electron transducer, and reduce the limitation on detection, since the carbon-based glycans would otherwise be insulating [42]–[44]. AuNPs were synthesized according to a published procedure [45]. Hydrogen tetrochloroaurate (III) trihydrate aqueous solution (1 mM, 50 mL) was stirred while on a hotplate. Once vigorous reflux was achieved, the solution was slowly titrated with 5 mL of 38.8 mM sodium citrate until the yellow solution became deep red. AuNPs have been previously characterized in terms of size, spectroscopic properties, and magnetic profile [46].

### E. EAM Polyaniline Nanostructure Synthesis

Magnetic/polyaniline core/shell nanoparticles were synthesized by polymerization of aniline monomer around gamma iron (III) oxide (γ-Fe_2_O_3_) cores [47]. Commercially purchased γ-Fe_2_O_3_ nanoparticles were dispersed in 50 ml of 1M HCl, 10-ml DI water, and 0.4-ml aniline monomer by sonication at 0 °C for 1 h. The γ-Fe_2_O_3_: monomer weight ratio was fixed at 1:0.6. 1 g ammonium persulfate in 20-ml DI water was added drop-wise as oxidant while the mixture was stirred at 0 °C. Color change from rust brown to dark green indicated formation of electrically active green polyaniline over the smaller brown γ-Fe_2_O_3_ nanoparticles. The reaction continued for 4 h with continuous stirring at 0 °C. The solution was filtered, washed with 1M HCl, 10% methanol, and diethyl ether, and dried for 18 h. The resulting green solid was ground into fine powder and stored in a vacuum desiccator. The electrically active magnetic/polyaniline c/s NPs have been previously characterized in terms of structure, size, magnetization, and conductivity [18], [19].

### F. EAM Immunofunctionalization

Desiccated EAM polyaniline nanoparticles were dissolved in 100 mM phosphate buffer (pH 7.4) to obtain a concentration of 10 mg/ml and sonicated for 15 min. The EAM polyaniline nanoparticles were then conjugated with α-H5 monoclonal antibodies by direct physical adsorption as described and confirmed by Pal and Alocilja [18]. α-H5 monoclonal antibody IgG2 (mouse ascites fluid) was added to the EAM polyaniline nanoparticles to obtain an antibody: EAM ratio of 1:10 by volume. The solution was incubated for 1 h at 25 °C in a rotational hybridization oven (Amerex Instruments, Inc., Lafayette, CA). Following adsorption of antibody, the immunofunctionalized nanoparticles were magnetically separated to remove any unbound antibody in the supernatant. The EAM-antibody complexes were washed twice with blocking buffer consisting of 100 mM tris–HCl buffer (pH 7.6) with 0.1% (w/v) casein, and resuspended in 100 mM phosphate buffer (pH 7.4). The EAM-antibody complexes were stored at 4 °C until use.

### G. SPCE Modification

SPCE sensors were washed with sterile DI water prior to modification and air dried. As described in Lin et al. [42], 25 μl of 2.5 mM glutaraldehyde solution were applied to the working area, incubated at 4 °C for 1 h, washed with DI water, and air dried. 25 μl of AuNP solution were applied to the glutaraldehyde-treated working electrode, incubated at 4 °C for 1 h, and washed with DI water. 25 μl of streptavidin at 1 μg/ml were applied to the working area and dried at 25 °C for 2 h.

### H. Capture Experiments

The SPCE sensors prepared with glutaraldehyde, AuNPs, and streptavidin were then treated with glycans, H5, and EAM-antibody complexes, in a step-wise fashion. 25 μl of the particular glycan concentration were added to the working area of the electrode and allowed to incubate at 25 °C for 30 min. Excess was rinsed with DI water and air dried at 25 °C for 10 min. Available sites were blocked with sequential additions of 25 μl Avidin D and biotin solutions, with DI water rinse and air dry after each. 25 μl of each H5 concentration were added, incubated for 30 min, rinsed, and dried. 100 μl of EAM-antibody complex solution were added to the electrode, allowed to react for 30 min, rinsed, and dried.

### I. Biosensor Testing Apparatus

Cyclic voltammetric measurements were performed using a 263 A potentiostat/galvanostat (Princeton Applied Research, MA, USA) connected to a personal computer. Data collection and analysis were controlled through the PowerSuite electrochemical software operating system (Princeton Applied Research, Wellesley, MA). SPCE sensors purchased from Gwent Inc., (U.K.) are shown in Fig. 1.

### J. Detection and Data Analysis

100 μl of HCl were applied to the SPCE electrode area and allowed to incubate for 5 min. The SPCE electrodes were connected to the potentiostat and cyclic voltammetry was performed at a scan rate of 55 mV/sec and a cyclic scan range of −0.4 to 1 V. The redox transition of polyaniline typically occurs within the potential range −0.2 to 0.4 V and is visible at scan rates ranging from 20 to 200 mV/s [48]. The scan range was chosen for the redox switching of polyaniline to be adequately observed and the scan rate was chosen for its intermediate value. Four consecutive 2-min scans were then recorded. The third scan was chosen for analysis based on previous experimentation that illustrated that the third scan produced the most pronounced current flow differences for different samples. For each experiment, three replications were performed. Several negative controls and blanks were also repeated in triplicate. The samples were calibrated against a negative control that consisted of the EAM-antibody application step alone. The total charge transferred Δ*Q* was computed from the cyclic voltammogram as the integral of current, according to the relationship

$$I = {{\Delta Q} \over {\Delta t}} \eqno{\hbox{(1)}}$$

where *I* = current (in A), Δ *Q* = charge transferred (in C), and Δ *t* = time elapsed (in s). Standard deviations and mean Δ *Q* values of the third scans for each dataset were calculated.

## III. Results and Discussion

**Fig. 2.**
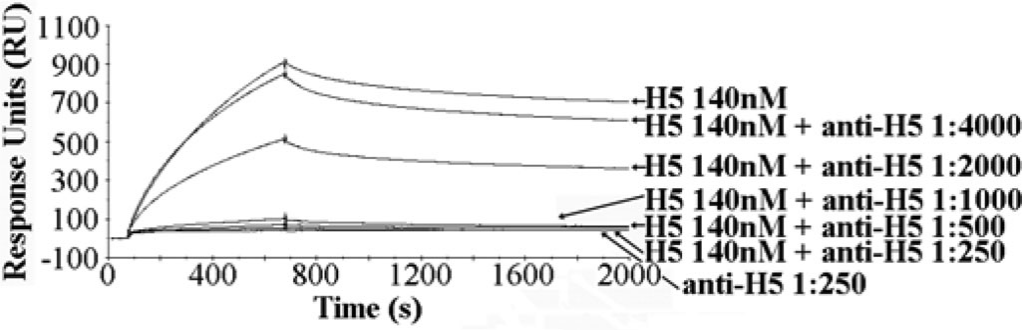
H5 Neutralization by α-H5 monoclonal antibody titration. H5/glycan binding is neutralized by α-H5 monoclonal antibody 1:500.

**Fig. 3.**
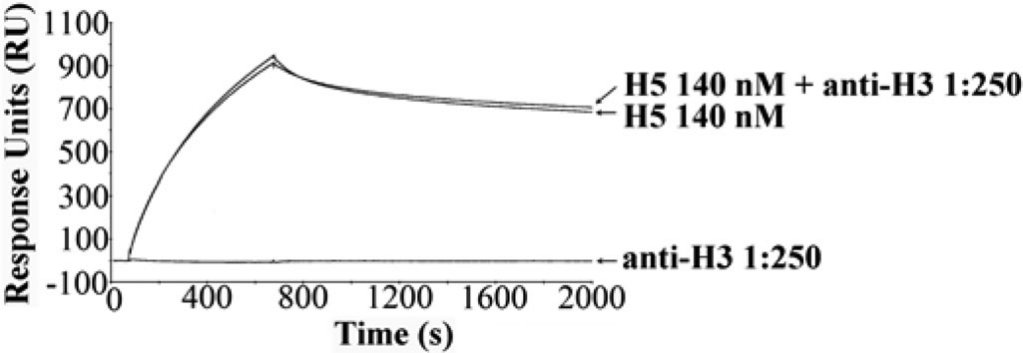
Preincubation of H5 with α-H3 monoclonal antibody does not inhibit H5/glycan binding.

The SPR assay demonstrated specific binding between H5-specific glycan receptors and recombinant H5, with high avidity. This binding can be neutralized when H5 is preincubated with neutralizing α-H5 monoclonal antibody and then injected over the glycan chip surface. The binding response of H5 at 140 nM was significantly decreased by α-H5 monoclonal antibody IgG2 (mouse ascites fluid) at 1:2000 dilution (see Fig. 2). α-H5 monoclonal 1:500 neutralized the H5/glycan binding at this H5 concentration, indicating that the HA binding inhibition displays a reproducible dose response. The HA/glycan binding was not inhibited by α-H3 monoclonal antibody 1:250, indicating no cross reactivity (see Fig. 3).

**Fig. 4.**
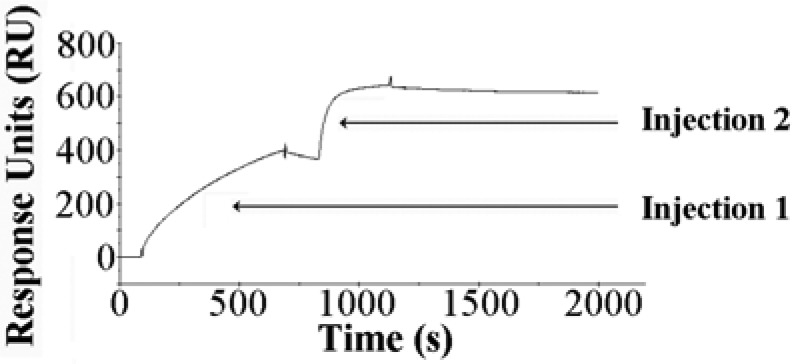
Antibody testing on Biacore. First injection: H5 at 140 nM for 10 min at 5 μl/min. Second injection: α-H5 monoclonal antibody (1:500) for 5 min at 5 μl/min.

Interestingly, addition of the same α-H5 monoclonal antibody following H5/glycan binding did not neutralize the H5/glycan binding but instead allowed a glycan/H5/α-H5 monoclonal antibody complex to form (see Fig. 4). The order of binding is thus important. As seen in the SPR neutralization experiment, preincubation of α-H5 monoclonal antibody with H5, followed by glycan addition, prevents H5/glycan binding because the α-H5 monoclonal antibody binds within, or otherwise blocks, the glycan receptor binding domain on H5. When the H5 and glycan are allowed to interact first, subsequent addition of α-H5 monoclonal antibody did not displace the glycan. Rather, the α-H5 monoclonal was able to bind the H5 either outside of the receptor binding domain or in an available receptor binding domain on H5 trimer or aggregate, as we suspect this HA preparation is composed of large aggregates. This α-H5 monoclonal antibody is thus appropriate for use in both the SPR neutralization assay as well as the biosensor sandwich-type assay.

**Fig. 5.**
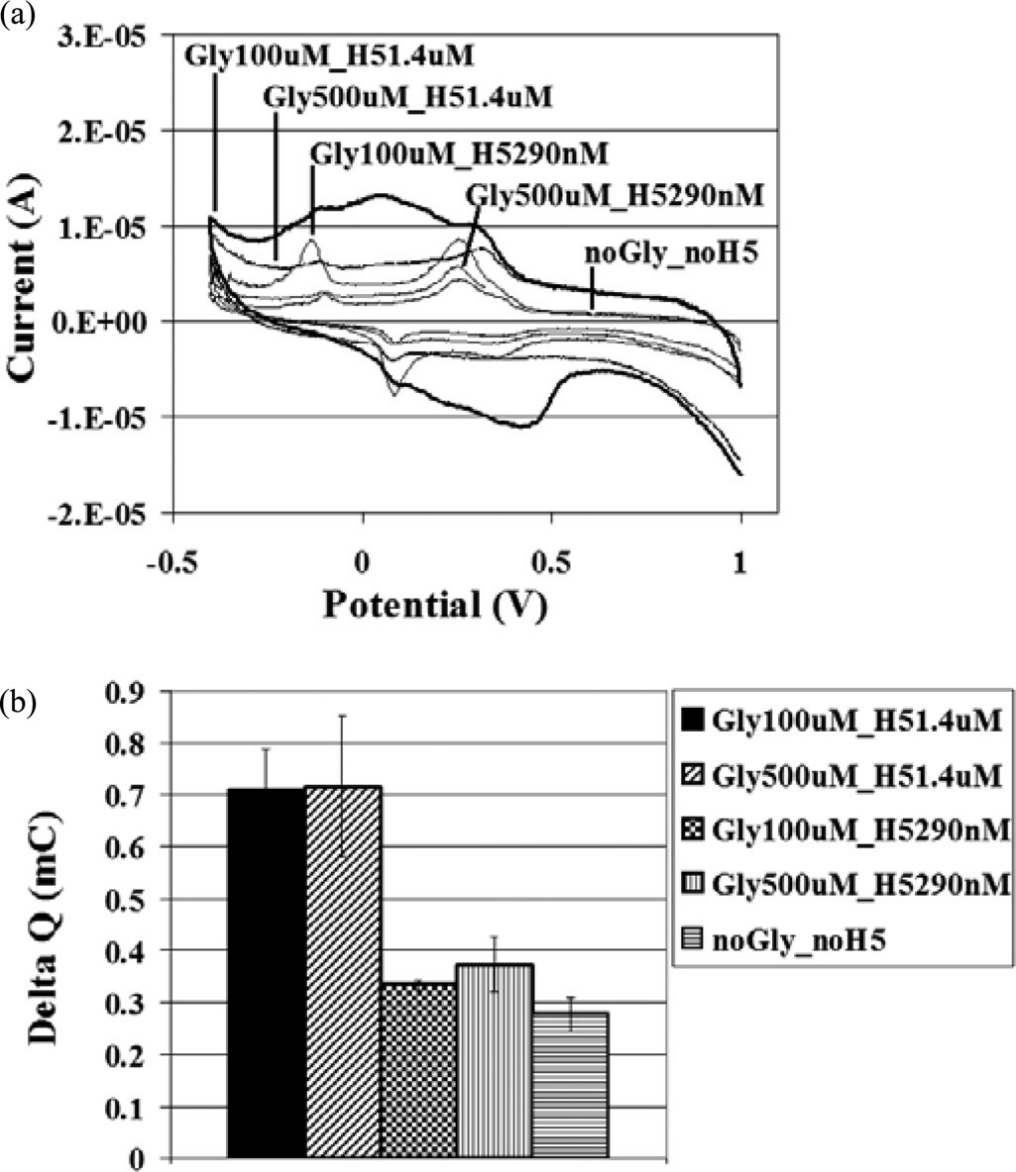
Glycan and H5 concentration studies. (a) Cyclic voltammograms of H5-specific glycan 100 μM and H5 1.4 μM; H5-specific glycan 500 μM and H5 1.4 μM; H5-specific glycan 100 μM and H5 290 nM; H5-specific glycan 500 μM and H5 290 nM; and no glycan and no H5, and (b) for the respective samples, mean Δ*Q* ± SD, *n* = 3 (SD = standard deviation, *n* = number of replicates).

**Fig. 6.**
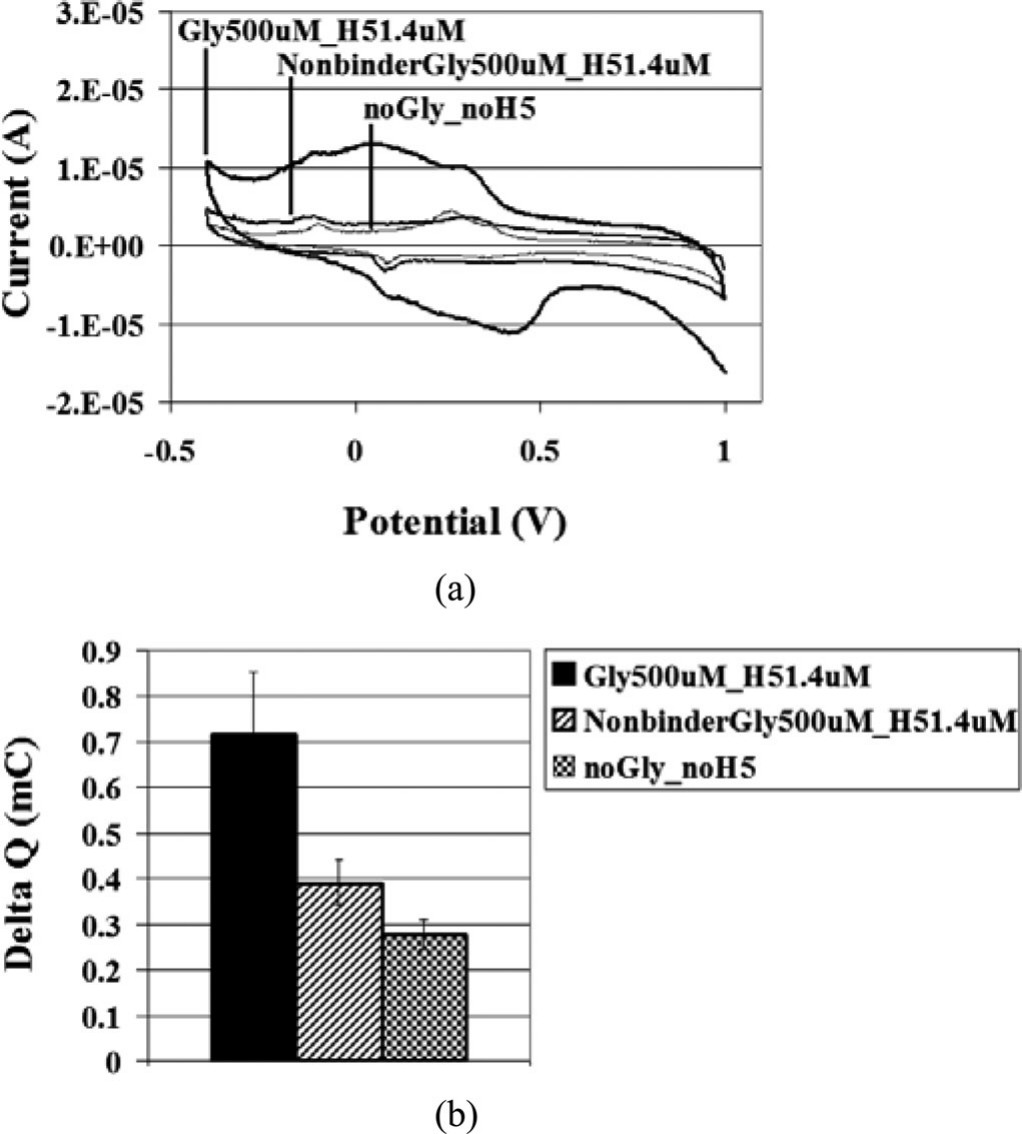
Specificity of binding between H5 and glycans. (a) Cyclic voltammograms of H5-specific glycan 500 μM and H5 1.4 μM; nonbinder (not H5-specific) glycan 500 μM and H5 1.4 μM; and no glycan and no H5, and (b) for the respective samples, mean Δ*Q* ± SD, *n* = 3 (SD = standard deviation, *n* = number of replicates).

**Fig. 7.**
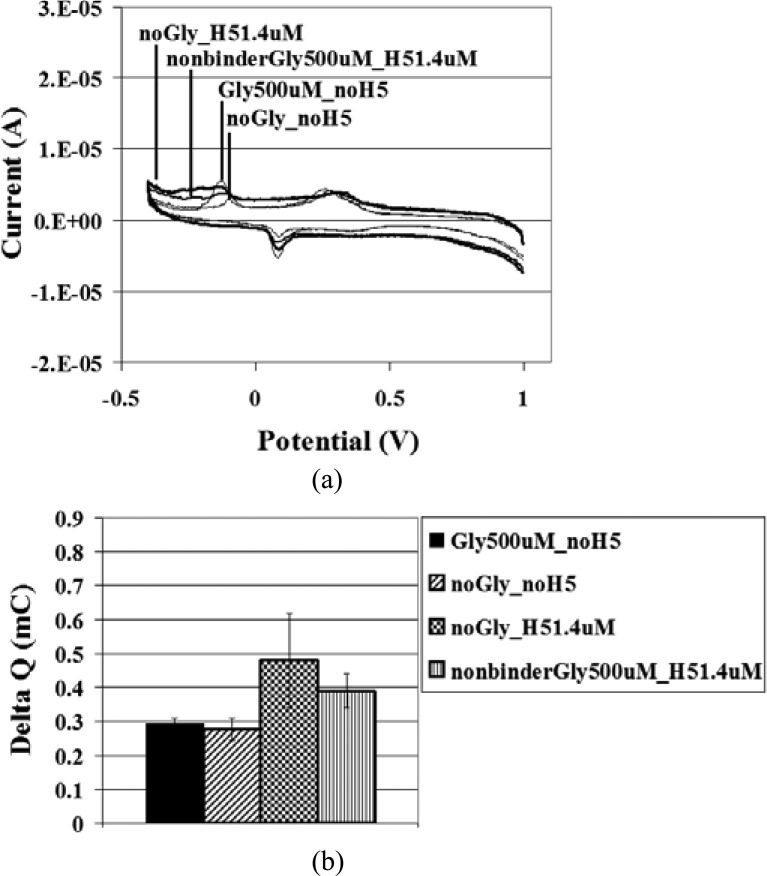
Negative control and blank evaluation. (a) Cyclic voltammograms of negative controls and blanks including no glycan and no H5; H5-specific glycan 500 μM and no H5; no glycan and 1.4 μM H5; and nonbinder (not H5-specific) glycan 500 μM and H5 1.4 μM, and (b) for the respective samples, mean Δ*Q* ± SD, *n* = 3 (SD = standard deviation, *n* = number of replicates).

The biosensor platform showed correlation to the SPR assay results. First, the appropriate glycan and H5 concentrations for the biosensor were explored. At the same H5 concentration, 1.4 μM, Δ *Q* remained consistent for glycan concentrations increasing from 100 to 500 μM (see Fig. 5). Figs. 5–7 show cyclic voltammograms of one replicate of each test for visual clarity, while the Δ*Q* reflects the average of the replicates. This may indicate that H5 at 1.4 μM did not saturate the glycan receptors at the higher glycan concentration. Alternatively, this could suggest that the prepared SPCE surface or streptavidin surface density was saturated at the lower glycan concentration of 100 μM, and that further addition of glycan did not yield any more available sites for H5 to bind. Keeping the glycan concentration constant at either 100 or 500 μM and decreasing the H5 concentration from 1.4 to 290 nM reduced the Δ*Q* to values near the negative control of no glycan and no H5. The samples with H5 at 290 nM were statistically similar for glycan at 100 and 500 μM, though statistically higher than the negative control (see Fig. 5). This could suggest that the glycan is the limiting reagent in terms of saturating the SPCE surface, but that when working with the glycan-saturated surface, the H5 becomes the limiting reagent. When the surface is saturated with glycans, the H5 concentration can be manipulated to obtain different readings. Interaction between the H5-specific glycan and H5 yielded a statistically higher Δ *Q* when compared to the interaction between the nonbinder (not H5-specific) glycan and H5, at the same concentrations (see Fig. 6). This demonstrates that the biosensor system shows specificity of binding.

A series of blanks were also investigated for comparison to the sample tests and to provide a background level of nonspecific binding. In Fig. 7, the negative control involved no glycan or H5 incubation steps, with only EAM-antibody complex incubation. The first blank included H5-specific glycan incubation, no H5 treatment, and EAM-antibody incubation. The second blank did not include glycan treatment, but included H5 and EAM-antibody incubation. The third blank included incubation with a nonbinder glycan, H5, and EAM-antibody. All negative controls and blanks were blocked with avidin and biotin after addition of glycan or, in the samples where no glycan was included, before addition of H5 or EAM-antibody. The absence of H5 yielded repeatable negative controls and blanks. Addition of H5 at 1.4 μM, in the presence of either no glycan or the nonbinder glycan, increased the Δ*Q*. However, these increased levels did remain below the Δ *Q* of the H5/H5-specific glycan binding. Because the presence of H5 in certain blanks resulted in higher Δ*Q* values than in those blanks and negative controls without H5, we can conclude that there may be low levels of nonspecific binding between H5 and the SPCE. This may be to the SPCE itself or to any of the immobilized partners, including glutaraldehyde, AuNPs, or streptavidin. We are currently exploring alternative or additional blocking techniques and reagents to decrease nonspecific binding. The lowest dilution of H5 that produced a signal distinguishable from the negative control of no glycan and no H5 as well as the blanks consisting of no glycan or nonbinder glycan binding to H5 1.4 μM was taken as the sensitivity of detection. Because the glycan and H5 dilutions were limited by reagent availability, this sensitivity is not a conclusive analytical sensitivity, but the detection limit for the experimental concentrations tested here. H5 at 1.4 μM was taken as the detection limit. This detection limit may be improved by a reduction of the nonspecific binding between H5 1.4 μM and the SPCE as well as by an assessment of dilutions between H5 1.5 and 290 nM.

**Fig. 8.**
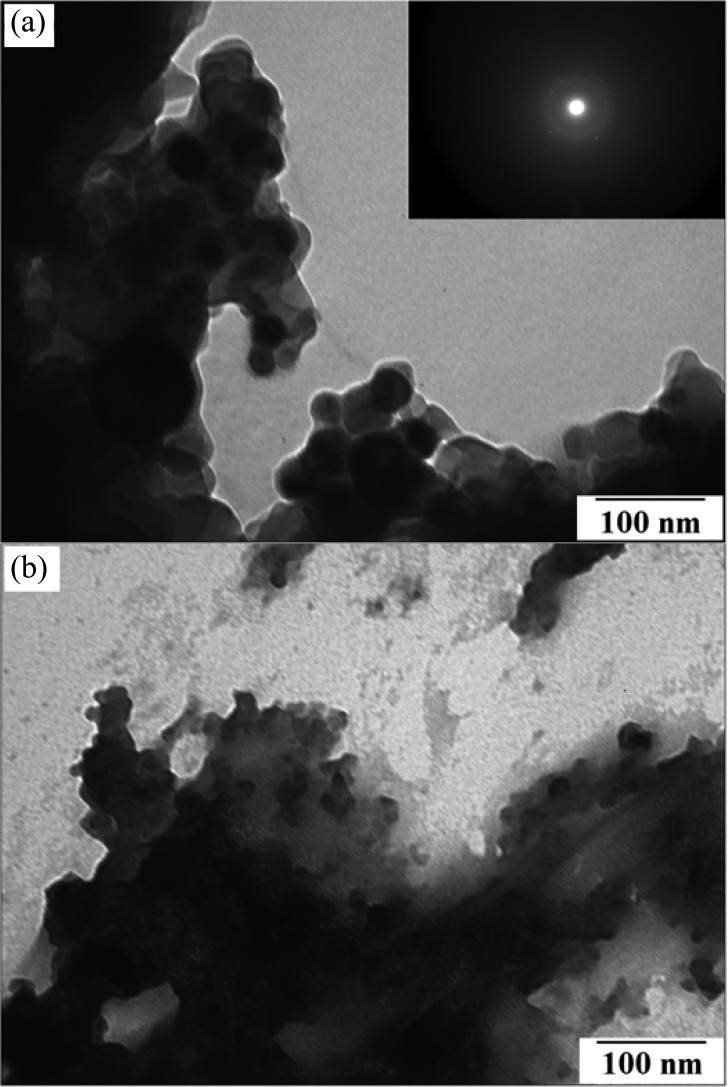
(a) TEM and electron diffraction micrograph of EAM polyaniline nanoparticles with gamma iron (III) oxide cores and (b) TEM of the EAM–α-H5 antibody conjugates.

**Fig. 9.**
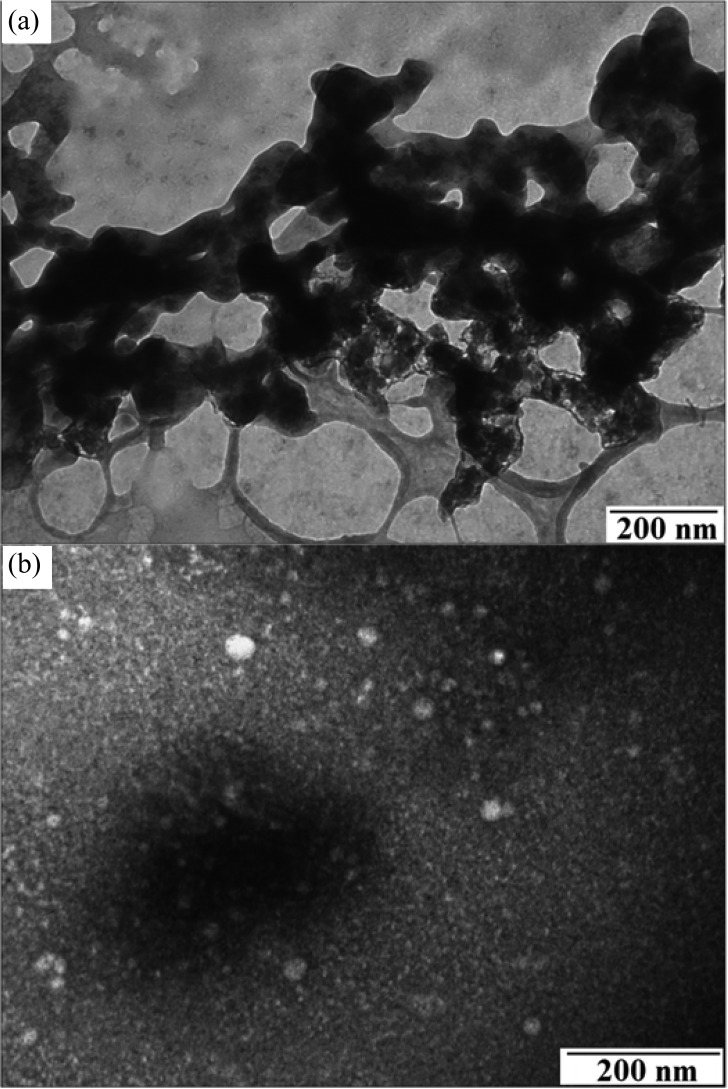
TEM of (a) synthetic glycans and (b) purified recombinant H5.

**Fig. 10.**
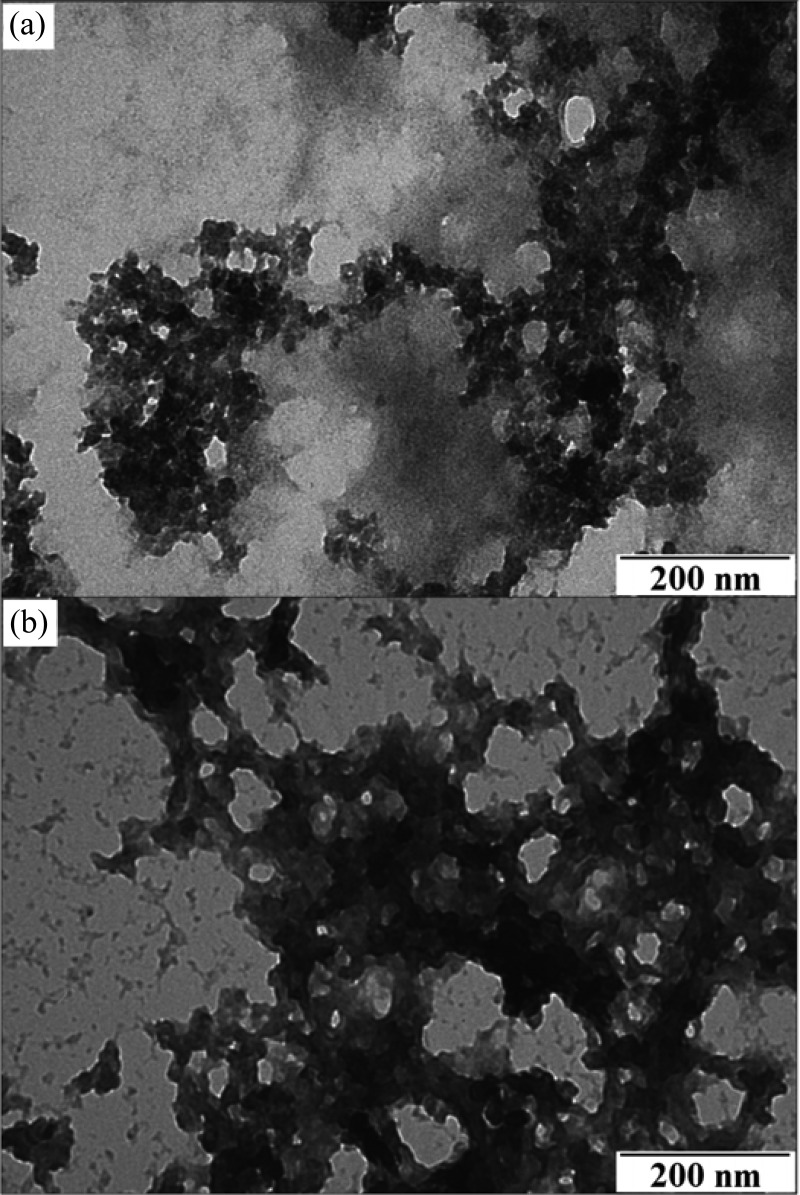
TEM of (a) glycan/H5/α-H5 monoclonal antibody complex as compared to (b) glycan/H5 complex.

The structural morphologies of the EAM polyaniline nanoparticles, glycans, and HA were analyzed by a JEOL, Peabody, MA, 100CX II transmission electron microscope (TEM). α-H5 antibody, glycans, and HA were stained with 1% uranyl acetate. The crystalline nature of the nanoparticles was studied by selected area electron diffraction using the JEOL 2200FS field emission TEM. Confirmation of polyaniline formation by TEM and FT-IR has been previously described in the literature and has not been repeated here [18], [49], [50]. As shown in Fig. 8(a), the TEM and electron diffraction micrograph revealed EAM polyaniline nanoparticle sizes in the 25–100-nm range. As shown in the TEM image, the γ-Fe_2_O_3_ cores are the darkest circular spots and are surrounded by the gray lighter colored polyaniline that is polymerized around the cores. TEM imaging of the EAM polyaniline nanoparticles immunofunctionalized with α-H5 antibodies revealed a rougher edge to the dark circular EAM nanoparticles, which could represent the bound antibody [see Fig 8(b)]. The synthetic glycan TEM revealed a structure characteristic of carbohydrate chains [see Fig. 9(a)]. The characterization of the recombinant H5 revealed aggregates of sizes varying from 13–35 nm, seen as white circular spots [see Fig. 9(b)]. This confirms our hypothesis that the H5 is in the form of large aggregates, since the influenza virion is approximately 100 nm in diameter and there are several hundred HA glycoproteins on the surface of each virion [6], [7]. H5 of 13–35 nm can be concluded to be a large aggregate. This may provide another reason for nonspecific binding between this “sticky” H5 and the SPCE surface. TEM imaging of the glycan/H5/α-H5 monoclonal antibody complex revealed a cloudier boundary as compared to the glycan/H5 with no antibody added [see Fig. 10(a) and (b)]. While the images do not offer definitive proof of antibody binding, the appearance of Fig. 10(a) does resemble the cloudy nature of the immunofunctionalized EAMs in Fig. 8(b), which could indicate that antibody was bound in both samples. If so, Fig. 10(a) and (b) would confirm the SPR binding results presented in Fig. 4.

Work is ongoing to further develop and optimize the biosensor in terms of saturating complementary reagent concentrations, incubation times, and detection procedures. Consistency of negative controls and blanks is under study with more satisfactory blocking techniques, to eliminate any nonspecific binding. We are currently exploring the ability of the biosensor to detect HA at concentrations that reflect the viral load in an infected patient; preconcentration of the analyte is one approach currently under investigation.

Our emerging technology is promising and novel due to its specificity, portability, and affordability. Applicability to detection of other FLUAV strains, including the current swine-origin H1N1, is also possible, as the biosensor is easily adaptable to any FLUAV strain once appropriate HA/glycan pairs are chosen. The Biacore SPR assay remains an appropriate technique for probing the specificity and avidity of such new relationships, and also for testing α-HA antibodies for cross-clade protection.

## IV. Conclusion

The SPR Biacore assay and biosensor platforms are complementary methods that have proven useful toward characterizing the binding specificity and avidity between synthetic glycan receptors and recombinant HA protein. This binding is essential for influenza viral infectivity. Specific antibody-mediated inhibition of H5/glycan binding may indicate that the binding observed on the Biacore system is physiologically relevant with α-H5 monoclonal antibody 1:500 neutralizing the H5/glycan binding at the H5 concentration of 140 nM. The biosensor is valuable as a rapid, specific, point-of-care detection method for identifying α2,6 specificity that will be useful to monitor animal influenza infections and to characterize pandemic potential in zoonotic strains. The biosensor demonstrated a detection limit for H5 of 1.4 μM and specificity of binding between glycan at 100 μM and H5 at 1.4 μM. EAM polyaniline nanoparticles can be consistently synthesized at the nanoscale. The development of such a biosensor technology which functions on the differential and specific binding of FLUAV HA to host SAs is a significant initiative with regard to disease monitoring and homeland security. Ultimately, the biosensor will be used for on-site serum or respiratory secretion testing. Our research will transition to detection of whole or pseudotyped virus in complex matrices, in which system the immunomagnetic separation property of the EAM-antibody complexes will be exploited. Future research will focus on improving biosensor system repeatability and sensitivity, as well as applicability to emerging FLUAV strains, including the swine-origin H1N1 strain.
